# CSF protein variations correlates with CSF oscillations in hydrocephalus patients

**DOI:** 10.1186/2045-8118-12-S1-O34

**Published:** 2015-09-18

**Authors:** Olivier Balédent, Vincent Puy, Jadwiga Zmudka, Cyrille Capel, Roger Bouzerar, Jérome Ausseil

**Affiliations:** 1 University hospital of Piardy Jules Verne, Amiens, France; 2 BioFlowImage, University of Piardy Jules Verne, Amiens, France; 3 General Hospital, Saint Quentin, France; 4 Inserm U1088, France

## Introduction

There are no previous reports comparing CSF biochemistry and its flow dynamics. As suggested by Milhorat, the complex system of CSF circulation through the brain may help to maintain a balance between the CSF flow rate and the CSF biochemical composition between different compartments. We hypothesize that this equilibrium may be disturbed in hydrocephalus patients and by using flow-MRI and CSF biological assessment, we could determine if alterations of CSF flow found in hydrocephalus patients affects their CSF biochemistry.

## Methods

9 hydrocephalus patients, 73 ± 8 years old, underwent a morphological MRI in which two CSF flow acquisitions were added to quantify CSF stroke volume in the aqueduct and in the spinal canal. A dynamic CSF index (Dynindex) was calculated equal to the product of the two CSF stroke volumes and to the cardiac cycle duration.

All patients had a CSF tap test insertion of a shunt. During these operations, CSF was collected from both the ventricular and spinal compartments. From these CSF samples, standard biochemical measurements were performed including chlorine, glucose and protein levels. For each CSF analysis, a biochemical ratio (Biochratio) was defined by divided the ventricular concentration by the spinal concentration.

## Results

The ventricular and spinal CSF stroke volumes were heterogeneous among the 9 patients. In comparison with previous normal aging investigations, stroke volumes were respectively in the aqueduct and the spinal compartments: diminished (n= 1, n = 1); normal (n=3, n=4) and increased (n=5, n=4) . The Dynindex varied from 0 to 100 with a mean value equal to 35. No difference between the ventricular and the spinal compartment was found for the concentrations of chlorine and glucose, and their Biochratio were respectively: 0,99±0.01 and 0,91±0.10. In contrast, the Biochratio of the protein demonstrated very different values amongst the population (from 1.21 and 5.58) and for each patient, Biochratios were strongly correlated (R=0.98, P<5.10-5) with the corresponding Dynindex.

## Conclusion

In the this small hydrocephalus population, CSF flow varied between the ventricles and the spinal compartments. Chlorine and glucose concentrations were equal in the ventricles and the spine, but protein concentration was different between the ventricles and spine and was highly correlated with the CSF flow oscillations.
